# Deciphering of Key Pharmacological Pathways of Poria Cocos Intervention in Breast Cancer Based on Integrated Pharmacological Method

**DOI:** 10.1155/2020/4931531

**Published:** 2020-10-09

**Authors:** Xiaoran Ma, Jibiao Wu, Cun Liu, Jie Li, Shixia Dong, Xiaolu Zhang, Jia Wang, Lijuan Liu, Xiaoming Zhang, Peng Sun, Jing Zhuang, Changgang Sun

**Affiliations:** ^1^College of First Clinical Medicine, Shandong University of Traditional Chinese Medicine, Jinan 250014, Shandong, China; ^2^College of Traditional Chinese Medicine, Shandong University of Traditional Chinese Medicine, Jinan 250014, Shandong, China; ^3^Clinical Medical Colleges, Weifang Medical University, Weifang 261000, Shandong, China; ^4^Department of Oncology, Weifang Traditional Chinese Hospital, Weifang 261041, Shandong, China; ^5^College of Pharmacy, Shandong University of Traditional Chinese Medicine, Jinan, China; ^6^Innovative Institute of Chinese Medicine and Pharmacy, Shandong University of Traditional Chinese Medicine, Jinan 250014, Shandong, China

## Abstract

**Objective:**

Poria cocos (Fuling), a natural plant, has recently emerged as a promising strategy for cancer treatment. However, the molecular mechanisms of Poria cocos action in breast cancer remain poorly understood.

**Methods:**

TCMSP database was used to screen the potential active ingredients in Poria cocos. GEO database was used to identify differentially expressed genes. Network pharmacology was used to identify the specific pathways and key target proteins related to breast cancer. Finally, molecular docking was used to validate the results.

**Results:**

In our study, 237 targets were predicted for 15 potential active ingredients found in Poria cocos. An interaction network of predicted targets and genes differentially regulated in breast cancers was constructed. Based on the constructed network and further analysis including network topology, KEGG, survival analysis, and gene set enrichment analysis, 3 primary nodes were identified as key potential targets that were significantly enriched in the PPAR signaling pathway.

**Conclusion:**

The results showed that potential active ingredients of Poria cocos might interfere with breast cancer through synergistic regulation of PTGS2, ESR1, and FOS.

## 1. Introduction

As one of the most common cancers in women, although screening and early detection significantly aid prevention and treatment, almost 1 in 4 newly diagnosed cancer cases in women are breast cancers [1, 2]. Over the past 30 years, breast cancer has become one of the fastest rising cancers in China, with an increase rate of nearly 96%, which is only slightly behind lung cancers [3]. Treatments of breast cancers include surgery, hormone therapy, radiotherapy, targeted therapy, and chemotherapy. However, all these treatments have adverse side effects or cause chemoresistance, which is encouraging researchers to look for new alternative therapies [4, 5]. Since some plants contain natural ingredients for the treatment of breast cancer, herbal therapy is being considered as a natural alternative.

Traditional Chinese medicine (TCM) from natural compounds has been developed since several years. TCM is not only used clinically in China and other Asian countries but also becoming increasingly popular in many developed countries like Australia and the United States [6]. The development of modern science and technology has greatly improved the acceptability, accessibility, and convenience of TCM. For example, artemisinin, extracted from *Artemisia annua*, is a Chinese herbal medicine used to treat malaria. Furthermore, single-target interventions may prove ineffective for complex systemic diseases like cancer with powerful biological networks [7]. Coincidentally, in many cases, TCM is used to treat diseases through multicompounds and multitargets [8]. Poria cocos (Fuling), a saprophytic fungus, has been classically used to prevent or treat various diseases. Poria cocos has diuretic, sedative, and nourishing effects on traditional Chinese medical science. Poria cocos has well-defined phytochemicals that mainly include polysaccharides, triterpenes, and steroids [9]. Recent studies have revealed that Poria cocos also possesses anti-inflammatory, proimmune, and antitumor effects [10]. In addition, pachymic acid present in Poria cocos can inhibit breast cancer cell invasion by downregulating MMP-9 expression [11]. Therefore, we speculate that Poria cocos may contribute to the treatment of breast cancer by regulating breast cancer-associated pathways, although the compound-target interaction spectrum and the specific mechanism remain unclear.

Network pharmacology is a new drug development method based on multipharmacology and network biology, which provides a valuable tool for extracting new targets for rational drug discovery [12]. Network pharmacology aims to study the complex and diverse relationships between drugs, targets, diseases, and pathways, which can solve problems in drug discovery research, such as drug resistance to single-target compounds and lack of drug efficacy [13]. Moreover, it can use bioinformatics and high throughput screening to construct a predictive “drug-target and disease” network model [14]. This relies on bioinformatics databases like Gene Expression Omnibus (GEO) to provide large amounts of raw data. GEO is a database supported by the National Center for Biotechnology Information (NCBI) of the United States, which has 1.3 million samples from more than 2889 organisms [15]. GEO database also contains information on miRNAs which are important regulators of intercellular communication. The miRNAs and noncoding RNAs regulate gene expression at the epigenetic level and also function in controlling cell survival, proliferation, and tissue development, thus, being pertinent in cancer progression [16]. Specific miRNAs can also help eliminate cancer cells and inhibit cancer metastasis [17].

In order to elucidate the comprehensive mechanism of action of Poria cocos intervention in breast cancer, we adopted the methods based on network pharmacology and bioinformatics, to screen for key network targets and pathway information. The results obtained were further validated by molecular docking. Our study hopes to provide insight into the Poria cocos mechanism of action and its use as a potential therapy for breast cancer.

## 2. Materials and Methods

### 2.1. Potential Active Components in Poria Cocos

We collected data regarding the chemical compounds present in Poria cocos from the Traditional Chinese Medicine Systems Pharmacology Database (TCMSP, http://lsp.nwu.edu.cn/), which is a unique systems pharmacology platform of Chinese herbal medicines and composed of a large number of entries with their absorption, distribution, metabolism, and excretion (ADME) properties. PubMed database was used for literature mining to prevent omitting of relevant compounds. We chose components that met the requirements of OB ≥ 30% and DL ≥ 0.18 as potential active ingredients of Poria cocos. The FDA defines OB (oral bioavailability) as “the rate and extent to which the active ingredient or active moiety is absorbed from a drug product and becomes available at the site of action” [18]. OB of the potential active components of Chinese medicine is one of the most important pharmacokinetic parameters in ADME, critical in drug discovery and development. DL (drug-likeness) refers to the similarities between ADME properties of components and that of known drugs.

### 2.2. Prediction of the Targets of the Potential Active Components in Poria Cocos

We used the TCMSP database and SwissTargetPrediction (http://www.swisstargetprediction.ch/) to obtain possible targets of potential active components in Poria cocos. All the potential active components were searched with PubChem (http://pubchem.ncbi.nlm.nih.gov/) to obtain their canonical SMILES strings. The canonical SMILES were then imported into SwissTargetPrediction to obtain targets of each potential active compound. We used UniProt KB database (http://www.uniprot.org/) to search for gene names of each protein target of active components that were obtained from TCMSP. Finally, we deleted the redundant targets of the same components obtained from the two databases to finally obtain 237 targets for the potential active ingredients of Poria cocos.

### 2.3. miRNA Data Extraction and Target Gene Prediction

We manually queried the GEO database (http://www.ncbi.nlm.nih.gov/geo/) for the keywords “breast cancer miRNA” and used the following criteria to filter the datasets: (a) the study used human samples; (b) data were from breast cancer tissues and nonbreast cancer tissues; and (c) sample size in each dataset was *n* > 20. Finally, GSE42128 dataset based on GPL15018 and GSE38867 dataset based on GPL15019 were selected, which included 76 tissue samples (49 breast cancer and 27 normal breast tissue samples). Furthermore, we used the GEO2R tool (http://www.ncbi.nlm.nih.gov/geo/geo2r/) to identify miRNAs differentially expressed in normal versus breast cancer tissues. Differentially expressed miRNAs were defined as having a relative expression change of |Log2FC| > 2 and *p* < 0.05. TargetScan (http://www.targetscan.org/vert_72/), miRWalk (http://mirwalk.umm.uni-heidelberg.de/), and miRDB (http://www.mirdb.org/miRDB/) were used to confirm the potential targets of miRNAs. High confidence targets that appeared at least twice among the 3 prediction software programs were analyzed further.

### 2.4. Verification of Overlapping Prediction Targets

To improve the accuracy of target gene prediction and reduce the rate of false positives, we used the GSE5764 dataset from the GEO database to further verify overlapping prediction targets. We used the GEO2R tool to identify differentially expressed genes (DEGs), with *p* < 0.05 and |Log2FC| > 2, from the confirmed datasets. We then shortlisted the common genes between the overlapping targets and DEGs, for further analysis.

### 2.5. Network Construction and Kaplan–Meier Test

Analyzing protein-protein interaction (PPI) networks provides a useful tool to evaluate the functions of proteins and interactions of genes. By setting the minimum interaction score to 0.4, the web-based STRING database (version 10.5) (http://string-db.org/) was used to produce PPI predictions of all genes that were targets of Poria cocos and the shortlisted DEGs in breast cancer, which were visualized using the network visualization software Cytoscape (http://cytoscape.org/, ver. 3.6.0). The topology scores of the nodes, including closeness centrality, betweenness centrality, and degree were measured using CentiScaPe plugin (version 2.2) and the top 5 genes were identified as hub genes. The Kaplan–Meier plotter (http://kmplot.com/analysis/), an online tool, enlists the effect of 54,675 genes on the survival of 10,461 cancer samples. We used the Kaplan–Meier plotter breast cancer mRNA database to assess the prognostic values of hub genes in breast cancer.

### 2.6. Gene Ontology and Pathway Enrichment

We performed Gene Ontology (GO) enrichment and pathway enrichment analyses using the online Database of Annotation, Visualization, and Integrated Discovery (DAVID) (https://david.ncifcrf.gov/). GO terms and enrichment pathways that had *p* < 0.05 were considered significant.

### 2.7. Gene Set Enrichment Analysis (GSEA)

GSEA is a computational method that is based on gene sets and establishes a database of molecular characteristics based on the known information about gene characteristics, location, and biological functions. The correlation between the key potential genes and biological mechanisms was analyzed with GSEA v3.0 (http://www.broad.mit.edu/gsea/) using the gene sets from the Molecular Signatures Database (MSigDB) as a reference. Enrichment results with nominal *p* < 0.05 and FDR value < 0.25 were considered statistically significant.

### 2.8. Molecular Docking Simulation

The SYBYL-X software is a powerful and efficient tool that verifies the binding ability of candidate targets to potential active components from herbs. We used SYBYL-X to implement molecular docking simulations. Crystal structure data of key targets were obtained from the RCSB Protein Data Bank, and the small molecule structures of the potential active components were obtained from the PubChem Project. Targets with molecular docking scores >6.0 were considered significant.

## 3. Results

### 3.1. Identification of Potential Active Components of Poria Cocos and Their Target Prediction

**Figure 1:**
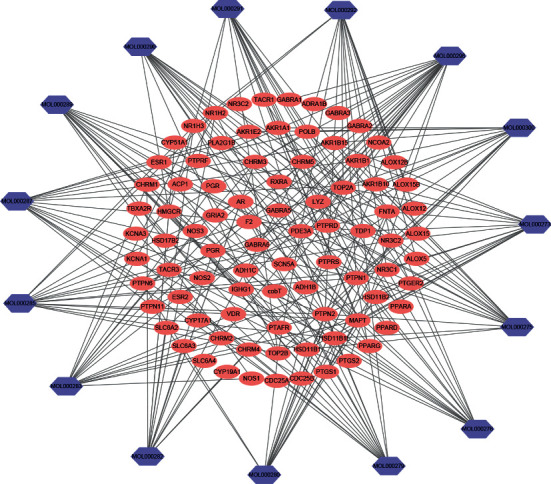
Compound-compound target network (blue hexagons represent compounds contained in Poria cocos; pink circular represents compound targets).

In total, 15 potential active components (Supplementary Table 1) from Poria cocos were selected from the TCMSP database, which met the criteria of OB ≥ 30% and DL ≥ 0.18. From the TCMSP and SwissTargetPrediction databases, we obtained potential targets for all 15 active components. After removing redundant targets of the same components between the two databases, 237 prediction targets (Supplementary Table 2) were identified for 15 potential active components of Poria cocos. Component-component target network was constructed by Cytoscape 3.6 (Figure 1).

### 3.2. Validation of Differentially Expressed Genes in Breast Cancer

GSE42128 dataset included 28 breast cancer tissue samples and 20 normal breast tissue samples, while GSE38867 dataset included 21 breast cancer tissue samples and 7 normal breast tissue samples. We found 781 differentially expressed microRNAs in GSE42128, including 588 upregulated and 193 downregulated microRNAs; and 43 differentially expressed microRNAs in GSE38867, including 27 upregulated and 16 downregulated miRNAs. We further analyzed 24 differentially expressed miRNAs that were common between the two datasets. Target genes of the 24 selected microRNAs were predicted and genes that appeared in at least two databases were selected (Supplementary Table 3), which were to be cross-referenced with the GSE5764 differentially expressed genes. Finally, 124 differentially expressed genes were obtained for further analysis (Supplementary Table 4).

### 3.3. PPI Network Construction and Survival Analysis

**Figure 2:**
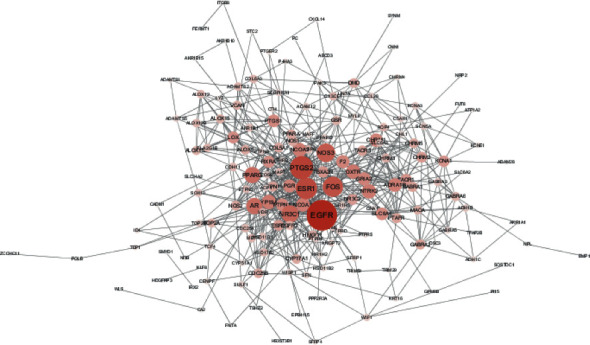
PPI network (deeper colors represent higher node connectivity).

**Figure 3:**
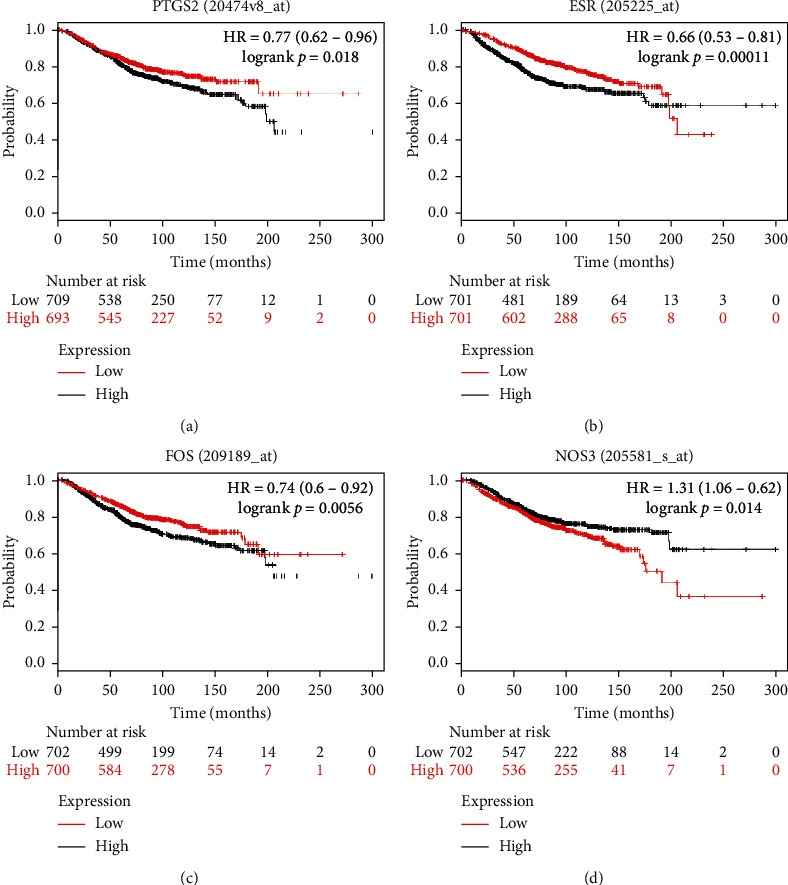
Analysis of survival curves of 4 hub genes. (a) PTGS2, (b) ESR1, (c) FOS, and (d) NOS3.

We generated a PPI network of common genes that were targets of Poria cocos active components and differentially expressed in breast cancer (Figure 2), which contained 163 nodes and 603 edges. Among these genes, the top 5 genes, including EGFR, PTGS2, ESR1, FOS, and NOS3, were defined as hub genes. The Kaplan–Meier plotter was used to investigate the prognostic values of the ten hub genes. The results showed that the abnormal expression of 4 hub genes (PTGS2, ESR1, FOS, and NOS3) was associated with unfavorable overall survival of breast cancer patients (Figure 3).

### 3.4. Gene Ontology and Pathway Enrichment

**Figure 4:**
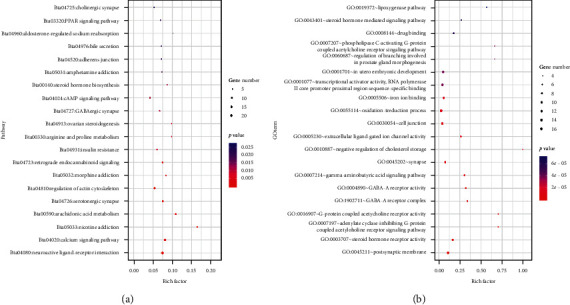
Bubble chart of the pathway enrichment and Gene Ontology. (a) KEGG pathway analysis and (b) GO analysis. The cutoff criterion was *p* < 0.05, and the first 20 pathways are shown.

To evaluate the contribution of all genes to the development of breast cancer, DAVID was used for functional enrichment analysis of all genes. These genes were extensively enriched in cancer-related pathways, including “PI3K-Akt signaling pathway,” “pathways in cancer,” and “PPAR signaling pathway” (Figure 4(a)). In addition, the functions of these genes including molecular function, biological processes, and cellular components were analyzed, and the results showed that these genes were enriched in various developmental and differentiation processes, such as “regulation of cell proliferation,” “positive regulation of cell growth,” and “positive regulation of epithelial cell proliferation” (Figure 4(b)).

### 3.5. Analysis of Key Target Characteristics

**Figure 5:**
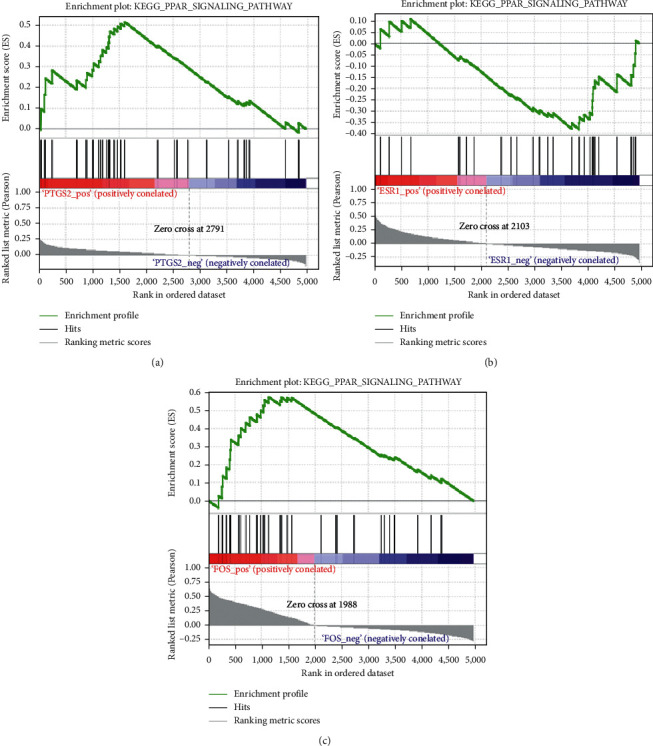
Identification of the enriched gene sets with GSEA analysis focused on a single gene as a phenotype in the merged microarray. PTGS2, ESR1, and FOS were associated with PPAR signaling pathways A, B, and C).

Based on GSEA, a single gene was used as phenotype for characterization analysis and enrichment results with nominal *p* < 0.05 and FDR value < 0.25 were considered statistically significant. The results show that PTGS2, ESR1, and FOS are significantly enriched in PPAR signaling pathway (Figure 5). Interestingly, when using DAVID to analyze the enrichment of all genes, the PPAR signaling pathway was also significantly enriched.

### 3.6. Molecular Docking

**Figure 6:**
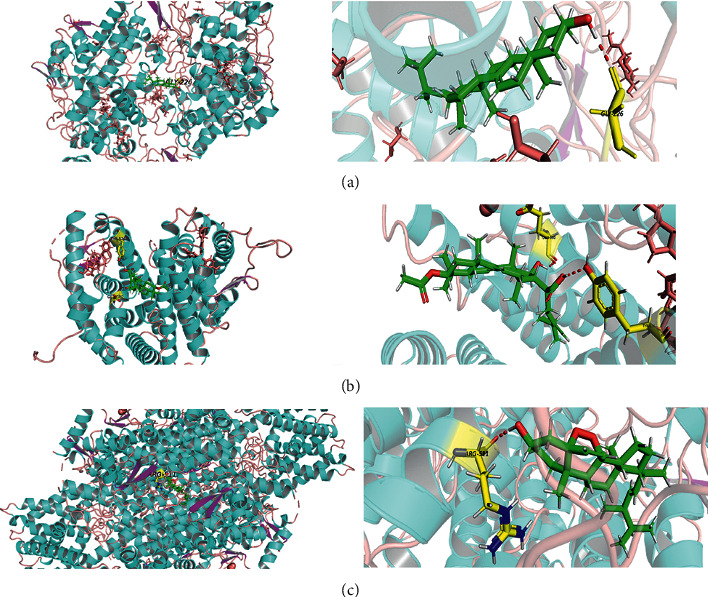
The protein-ligand of the docking simulation. (a) PTGS2 and stellasterol, (b) ESR1 and dehydropachymic acid, and (c)FOS and ergosterol peroxide.

In the previous step, we performed a single gene characterization of the hub gene related to the overall survival rate of breast cancer, and we found that all PTGS2, ESR1, and FOS were enriched in the PPAR signaling pathway. Therefore, these three genes were identified as key potential genes. We analyzed the docking potentials of these genes with 15 potential active components of Poria cocos using SYBYL-X (Supplementary Table 5). A total of 45 pairs of key potential genes (*n* = 3) and potential active components (*n* = 15) were used for docking. The docking scores of 15 pairs of potential active component-key potential gene interactions were greater than 6.0, indicating that they had strong binding free energy. In addition, our results also showed that the docking points of each potential target with at least one potential active ingredient were greater than 6.0. We further optimized the docking of specific protein-ligand interactions of 3 gene-compound pairs, with the highest docking scores using PyMoL (Figure 6).

## 4. Discussion

In most countries, breast cancer is the most commonly diagnosed cancer in women and is one of the leading causes of cancer-related deaths [1]. Initiation of breast cancer is related to not only tumor suppressive gene inactivation and oncogene activation but also stem cell disorders, abnormal immune functions, and cytokine imbalance [19]. The etiology of many complex diseases, including cancer, is dependent on multiple factors. There is growing evidence that cancers are unlikely to respond to modulations of a specific single target, and interventions at multiple points in the system are mandatory for successful treatment [7, 20]. In this study, we used the computational method of network pharmacology to identify potential active compounds in Poria cocos and identified the key points of interventions, rather than a single specific target.

Poria cocos, a commonly used traditional Chinese medicine, is considered as a promising candidate for alternative cancer therapy. Moreover, some active ingredients in Poria cocos have been shown to have anti-inflammatory, antiproliferative, and pain reduction effects. For instance, ergosterol peroxide inhibits the action of cyclooxygenase enzymes (COX) and reduces pain related to inflammation [21]. Pachymic acid and poricoic acid A, lanosterol triterpenoids from Poria cocos, show anti-inflammatory, anticancer, antivomiting, and cytotoxic effects, warranting the use of Poria cocos as a potential anticancer agent [22, 23]. Many other active components from Poria cocos could have potential antitumor effects, conforming to the synergistic effects of multiple ingredients of traditional Chinese medicine.

Network pharmacology is a multidisciplinary, highly informative field of research, that seeks to identify the key targets in disease-related networks through data mining, statistical methods, modeling, graph theory, and information visualization [24]. At the same time, it can predict targets in the networks to maximize damage to the required systems to treat complex diseases [25]. Excavating underlying information from multistage interactions, especially when dealing with big data, is distinctively advantageous for network analysis [26]. In addition, since most carcinogens exist within normal cells in the form of mutated proteins, the ideal cancer treatment is aimed at targeting the proteins or interactions essential in cancer cells, but not in normal cells. Network pharmacology can produce improved therapeutic indices and selective suppression of mutations by targeting differences in network topology [25]. Therefore, with the rapid development of systems biology, bioinformatics, and pharmacology, network-based drug discovery is deemed to be a more cost-effective and promising drug development strategy [27–29]. Moreover, in modern drug development, protein-protein or protein-ligand docking plays a key role in predicting ligand-protein receptor or enzyme binding, using shape parameters and electrostatic interactions, to quantify the direction of interactions [30]. The purpose of molecular docking is to predict the best mode of matching between ligands and macromolecules [31].

Based on the constructed PPI network, we carried out pathway enrichment analysis and found that target genes were significantly enriched in PPAR signaling pathway. Interestingly, each key candidate target was characterized based on GSEA, and it was found that differentially expressed PTGS2, ESR1, and FOS were also significantly enriched in this pathway. One of the mechanisms of drug resistance in cancer cells involves the release of exosomes mainly through the transfer of drug transporter proteins and nucleic acids or accumulation of anticancer drugs [32]. Ceramide, regulated by PPAR signaling pathway, is a regulatory molecule associated with exosome secretion [33, 34]. In addition, recent studies have shown that the association between metabolic syndrome and cancer can be increased by activating different PPAR signaling pathways. For example, SG Vitale and Lagana discussed the important role of nuclear receptors in PPAR signaling pathway in tumorigenesis and metabolic disease development [35].

Poria cocos may play a therapeutic role in breast cancer by binding and regulating specific protein targets. We speculate that the top 5 nodes identified by topology analysis of the PPI network could be the hub targets for breast cancer treatment by Poria cocos. Further screening of the targets showed three targets of PTGS2, ESR1, and FOS as the key potential genes. PTGS2 was targeted by 2 potential active compounds from Poria cocos–pachymic acid and hederagenin. PTGS2, a polymorphic gene, encodes cyclooxygenase-2 (COX-2) which is overexpressed in breast cancer and is related to invasive parameters such as tumor size, angiogenesis, and low overall survival [36, 37]. In addition, COX-2 is considered a biomarker for risk stratification of atypical hyperplasia of breast cancers and promotes the progression of ductal carcinoma in situ (DCIS) to invasive breast cancer, chemical resistance, and osteolytic bone metastasis of breast cancer cells [38–41]. Estrogen is a considerable regulator of mammary gland growth and differentiation, and it is also key in the occurrence and development of breast cancer [42]. Furthermore, studies have shown that excessive endogenous and endogenous estrogens may lead to pathological changes in many cancer cell lines, including breast cancer cells [43]. Therefore, as a gene encoding estrogen receptor, the genetic variation of ESR1, may expose the potential risk of breast cancer. FOS gene is a member of the immediate-early (IE) response gene family and FOS protein is a part of the activating protein-1 (AP-1) transcription factor complex. FOS interacts with JUN protein family members to mediate transcriptional activation and plays a key role in breast cancer [44, 45]. In addition, FOS is involved in several cellular processes related to carcinogenesis including proliferation, apoptosis, invasion, migration, metastasis, and angiogenesis [46–48]. FOS can also drive tumor transformation and malignant progression by regulating a variety of tumor-related target genes.

In addition, our results identified five miRNAs including miR-490-5p, miR-3200-5p, miR-3666, miR-338-5p, and miR-3925-5p associated with key potential genes. miR-490-5p is downregulated in bladder cancers, renal cell carcinoma, and liver cancers [49–51]. Overexpression of miR-490-5p in T24 bladder cancer cells significantly reduced the expression of c-FOS, inhibiting cell proliferation and invasion, and inducing cell apoptosis [52]. In locally advanced breast cancer, the expression of miR-3200 positively correlated with tumor differentiation and negatively correlated with the clinical stage [53]. miR-3200-5p is a key factor that enhances the invasiveness of osteosarcoma cells by regulating the level of BRMS1 protein [54]. The effect of miR-3666 on cell growth is potentially caused by changes in cell proliferation rather than alteration of apoptosis [55]. miR-3666 targets Sirtuin 7 to inhibit the growth of non-small cell lung carcinoma cells, by promoting the apoptotic signal transduction pathway [56]. Abnormal expression of miR-338-5p induces cell apoptosis and inhibits cell proliferation in many tumor types such as gastric cancer, esophageal cancer, and glioma [57–60]. Furthermore, there are nearly no relevant studies for other malignancies, and previous reports concerning the relationship of miR-3925-5p to breast cancer are lacking. However, previous reports on four other miRNAs also show that our results are reliable and valuable.

The current research combines network pharmacology and bioinformatics methods to clarify the molecular and pharmacological mechanism of Poria cocos action against breast cancer, from a systematic perspective. Although further experiments are needed to demonstrate the accurate action (e.g., activation or inhibition) of the potential compounds on key potential genes, it provides a significant basis for further optimizations of experimental design for mechanistic studies.

## 5. Conclusion

In summary, these biological studies further confirmed the reliability of our research model in exploring the mechanism of Poria cocos in the treatment of breast cancer. Potential active ingredients of Poria cocos may interfere with breast cancer through synergistic regulation of key genes (PTGS2, ESR1, and FOS) that participate in the PPAR signaling pathway.

## Data Availability

The data used to support the findings of this study are included within the supplementary information files.

## Supplementary Materials

Supplementary Materials Supplementary Table 1: information on key compositive compounds of Poria cocos. Supplementary Table 2: prediction of putative targets for compositive compounds of Poria cocos. Supplementary Table 3: prediction of putative targets for the selected microRNAs of breast cancer. Supplementary Table 4: differentially expressed genes in breast cancer. Supplementary Table 5: detailed information on molecular docking of compounds.
